# Cyr61 promotes epithelial-mesenchymal transition and tumor metastasis of osteosarcoma by Raf-1/MEK/ERK/Elk-1/TWIST-1 signaling pathway

**DOI:** 10.1186/1476-4598-13-236

**Published:** 2014-10-19

**Authors:** Chun-Han Hou, Feng-Ling Lin, Sheng-Mon Hou, Ju-Fang Liu

**Affiliations:** Department of Orthopedic Surgery, National Taiwan University Hospital, Taipei, Taiwan; Department of Dermatology, Sijhih Cathay General Hospital, Taipei, Taiwan; Department of Orthopedic Surgery, Shin-Kong Wu Ho-Su Memorial Hospital, NO. 95 Wen Chang Road, Taipei, Taiwan; Central Laboratory, Shin-Kong Wu Ho-Su Memorial Hospital, NO. 95 Wen Chang Road, Taipei, Taiwan

**Keywords:** Osteosarcoma, Cyr61, EMT, Migration

## Abstract

**Background:**

Osteosarcoma is the most common primary malignant tumor in children and young adults, and its treatment requires effective therapeutic approaches because of a high mortality rate for lung metastasis. Epithelial to mesenchymal transition (EMT) has received considerable attention as a conceptual paradigm for explaining the invasive and metastatic behavior during cancer progression. The cysteine-rich angiogenic inducer 61 (Cyr61) gene, a member of the CCN gene family, is responsible for the secretion of Cyr61, a matrix-associated protein that is involved in several cellular functions. A previous study showed that Cyr61 expression is related to osteosarcoma progression. In addition, Cyr61 could promote cell migration and metastasis in osteosarcoma. However, discussions on the molecular mechanism involved in Cyr61-regulated metastasis in osteosarcoma is poorly discussed.

**Results:**

We determined that the expression level of Cyr61 induced cell migration ability in osteosarcoma cells. The Cyr61 protein promoted the mesenchymal transition of osteosarcoma cells by upregulating mesenchymal markers (TWIST-1 and N-cadherin) and inhibiting the epithelial marker (E-cadherin). Moreover, the Cyr61-induced cell migration was mediated by EMT. The Cyr61 protein elicited a signaling cascade that included αvβ5 integrin, Raf-1, mitogen-activated protein kinase (MEK), extracellular signal-regulated kinase (ERK), and Elk-1. The reagent or gene knockdown of these signaling proteins could inhibit Cyr61-promoted EMT in osteosarcoma. Finally, the knockdown of Cyr61 expression obviously inhibited cell migration and repressed mesenchymal phenotypes, reducing lung metastasis.

**Conclusion:**

Our results indicate that Cyr61 promotes the EMT of osteosarcoma cells by regulating EMT markers via a signal transduction pathway that involves αvβ5 integrin, Raf-1, MEK, ERK, and Elk-1.

**Electronic supplementary material:**

The online version of this article (doi:10.1186/1476-4598-13-236) contains supplementary material, which is available to authorized users.

## Background

Osteosarcoma is the most prevalent primary malignant bone tumor that affects mainly children and adolescents. Osteosarcoma treatment has undergone dramatic changed drastically over the past 20 years, whatever the survival rate shows limited. Thus far, the 5-year survival rate is approximately 20% with surgical treatment alone. Moreover, half of the patients often exhibit pulmonary metastasis, which results in high patient mortality [1]. Thus, chemotherapy is typically employed in an adjuvant case for improving the prognosis and long-term survival. However, recurrence frequently manifests as pulmonary metastasis or, less frequently, metastasis to distant bones or as a local recurrence [2–4]. Thus, a novel strategy that would effectively inhibit metastasis, particularly to the lungs, from the primary osteosarcoma site is highly desirable.

Cancer metastasis is a critical step in tumor progression and a major cause of mortality in cancer patients. Epithelial to mesenchymal transition (EMT) has received considerable attention as a conceptual paradigm for explaining invasive and metastatic behavior during cancer progression [5]. EMT is a normal physiologic process in vertebrate development and tissue homeostasis, and is increasingly considered to be involved in disease states such as tissue fibrosis and cancer [6]. EMT is a process through which epithelial cells lose their polarity and are converted into a mesenchymal phenotype [5, 7]. A hallmark of EMT is the loss of epithelial characteristics, such as a decrease in the expression of the cell adhesion molecular E-cadherin and the acquisition of a mesenchymal phenotype accompanied by increased vimentin expression. EMT-related transcription factors such as TWIST-1, snail, slug, ZEB1, and ZEB2 orchestrate the EMT, and enable the early steps of metastasis, which consist primarily of local invasion and the subsequent dissemination of tumor cells to distant sites [8]. These transcription factors repress E-cadherin expression by binding to the E-box in the E-cadherin gene promoter, and consequently promote EMT [9–13]. Substantial evidence has indicated that osteosarcoma exhibits EMT-like states, characterized by changes in the expression of EMT-related transcription factors, such as TWIST-1, snail, and ZEBs, which are involved in the complex pathogenesis of osteosarcoma [14]. Targeting these transcription factors may provide a novel opportunity in osteosarcoma treatment by controlling metastasis.

Cysteine-rich 61 (Cyr61) is the first discovered member of the CCN family [15], which comprises Cyr61/CCN1, connective tissue growth factor (CTGF/CCN2), nephroblastoma overexpressed (Nov/CCN3), Wisp-1/elm1 (CCN4), Wisp-2/rCop1 (CCN5), and Wisp-3 (CCN6). All members of the CCN family share a common domain structure and exert variant cellular functions such as the regulation of cell division, chemotaxis, apoptosis, adhesion, motility, and ion transport [16–18]. The Cyr61 protein has been reported to mediate cell adhesion, stimulate chemostasis, augment growth factor-induced DNA synthesis, foster cell survival, and enhance angiogenesis [19, 20]. Previous studies have found that Cyr61 expression is associated with breast cancer, pancreatic cancer, and gliomas [21–24]. Downregulated Cyr61 expression has been reported in prostate cancer, uterine leiomyoma, rhabdomyosarcoma, and non-small-cell lung carcinoma [25–28]. The ambiguous expression of Cyr61 in different types of cancer suggests that Cyr61 may exert a sophisticated function depending on the cellular context.

Previous studies have shown that Cyr61 modulates cell migration and invasion in human cancer cells [29–31]. Moreover, Cyr61 has been proposed to play a pivotal role in osteogenesis and an aberrant expression of Cyr61 is associated with osteosarcoma progression and lung metastasis [32]. Nevertheless, whether Cyr61 promotes EMT in osteosarcoma remains unclear. This paper is the first to provide evidence that Cyr61 increases EMT in osteosarcoma and contributes to lung metastasis. In addition, Cyr61-promoted EMT is mediated by αvβ5 integrin, Raf-1, mitogen-activated protein kinase kinase (MEK), extracellular signal-regulated kinase (ERK), and Elk-1 signaling pathways, and may be involved in the regulation of EMT in osteosarcoma. Finally, we show that the knock-down of Cyr61 expression inhibits lung metastasis in osteosarcoma. In conclusion, our findings revealed that Cyr61 regulated EMT and thus promoted lung metastasis in osteosarcoma.

## Results

### Cyr61 overexpression was associated with high migration potential in osteosarcoma

**Figure 1:**
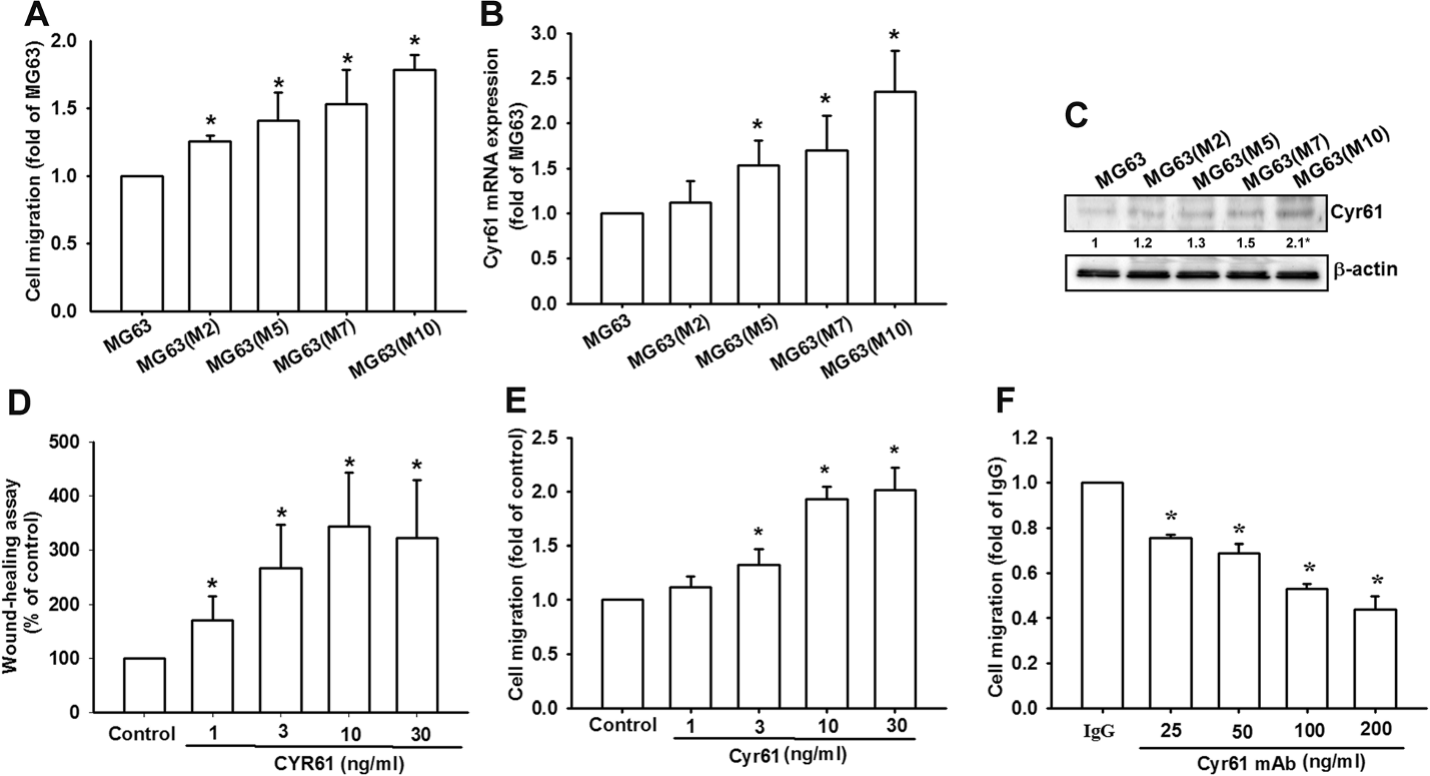
**Cyr61 is associated with the cell migration phenotype. (A)** MG63 cells and its sublines were selected according to description in the Methods section, and cell migration was examined using the Transwell assay. **(B and C)** Total mRNA and proteins were extracted from the MG63 cells and its sublines, and Cyr61 expression was detected using qRT-PCR and western blotting. **(D and E)** Cells were incubated with Cyr61 (1-30 ng/mL) for 24 h, and cell migration was measured using the Transwell and wound-healing assays. **(F)** MG63 cells were pretreated with anti-Cyr61 mAb (25-200 ng/mL) for 24 h. Cell migration was measured after 24 h using the Transwell assay. The results are expressed as the mean ± SEM of triplicate samples. *P <0.05 compared with the control group.

A previous study indicated that Cyr61 overexpression is correlated with a poor prognosis, and promotes metastasis in osteosarcoma [33]. However, the molecular mechanism involved in Cyr61-induced metastasis in osteosarcoma has not been thoroughly investigated. We clarified the correlation between Cyr61 and cell migration ability in osteosarcoma. We selected the high-migration-ability MG63 cell sublines by using the Transwell assay, and our results revealed that the mRNA and protein expression levels of Cyr61 increased in the high-migration-prone subline (MG63, M7, and M10) (Figure 1A-C, respectively). Moreover, treating osteosarcoma cells (MG63) with recombinant Cyr61 dramatically increased the cell migration ability, demonstrated using a migration assay and a wound healing assay (Figure 1D-E). Finally, pretreating MG63 cells with a Cyr61-neutralized antibody evidently abolished Cyr61-induced cell migration (Figure 1F). These results indicate that Cyr61 plays a pivotal role in osteosarcoma migration ability.

### Cyr61 regulated EMT markers and promoted mesenchymal transformation

**Figure 2:**
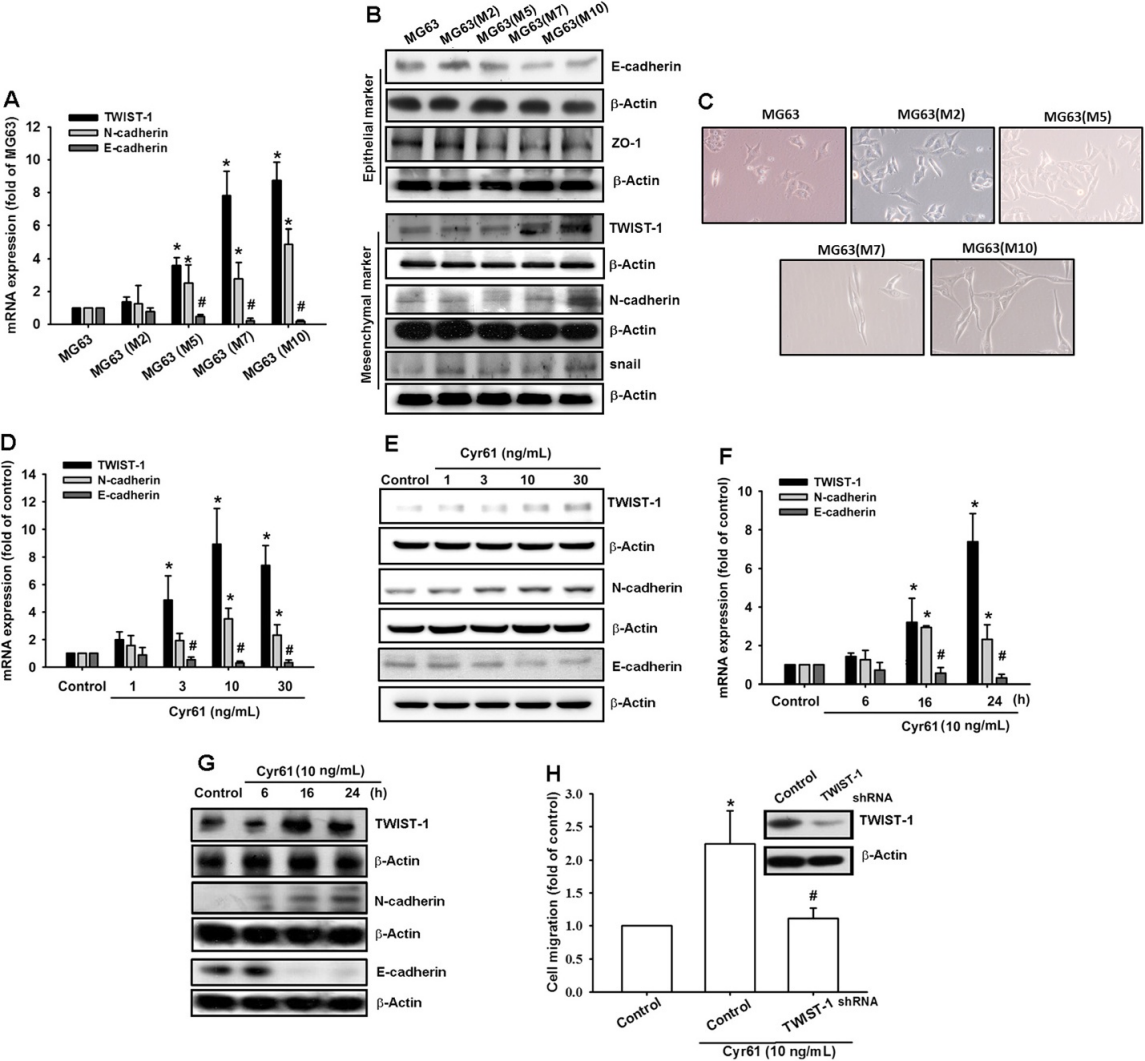
**Cyr61 regulates EMT markers and promotes mesenchymal transformation. (A and B)** Total mRNA and proteins were extracted from the MG63 cells and its sublines, and the expression levels of TWIST-1, N-cadherin and E-cadherin were detected using qRT-PCR and western blotting. **(C)** Phase-contrast images of the MG63 cells and their sublines were photographed. The subconfluent cultures were shown to indicate the morphological differences between MG63 and the sublines. **(D and E)** Cells were incubated with Cyr61 (1-30 ng/mL) for 24 h, total mRNA and proteins were extracted from the MG63 cells, and the expression levels of TWIST-1, N-cadherin, and E-cadherin were detected using qRT-PCR and western blotting. **(F and G)** Cells were incubated with Cyr61 (10 ng/mL) for different time (0-24 h), total mRNA and proteins were extracted from the MG63 cells, and the expression levels of TWIST-1, N-cadherin and E-cadherin were detected using qRT-PCR and western blotting. **(H)** MG63 cells were transfected with TWIST-1 shRNA or control shRNA for 24 h, followed by treatment with Cyr61 (10 ng/mL) for 24 h, and cell migration was analyzed using the Transwell assay. The results are expressed as the mean ± SEM of triplicate samples. *P <0.05 compared with the control group, and ^#^P <0.05 compared with Cyr61 treatment.

Previous studies have proposed that EMT is associated with cancer cell migration, tumor metastasis, and progression [7, 34]. To contribute to these observations, we investigated whether Cyr61-induced migration is regulated by the mesenchymal transformation of osteosarcoma. We compared epithelial and mesenchymal cell markers in MG63 cells and other sublines. The expression patterns of the EMT markers were found to be tightly correlated with Cyr61 expression in these sublines. The most migratory subline, MG63 (M10), expressed mesenchymal markers (N-cadherin and TWIST-1), but fewer epithelial markers (E-cadherin) (Figure 2A&B). Moreover, the most migratory subline MG63 (M10), showed a loose cell contact and spindle-shaped morphology representative of EMT, but not a cobblestone-like morphology in parental MG63 cells (Figure 2C). To clarify the role of Cyr61 in the EMT of osteosarcoma, the MG63 cells were treated with recombinant Cyr61. The results indicated that Cyr61 induced the expression of mesenchymal markers (N-cadherin and TWIST-1), but repressed an epithelial marker (E-cadherin) in a time- and dose-dependent manner (Figure 2D-G). Finally, we used the TWIST-1 short hairpin RNA (shRNA) to demonstrate whether Cyr61-induced cell migration is mediated by TWIST-1 upregulation, and the results revealed that transfection with TWIST-1 shRNA dramatically inhibited Cyr61-induced cell migration in osteosarcoma (Figure 2H). These results revealed that Cyr61 could promote cell migration by promoting the mesenchymal transformation of osteosarcoma.

### Cyr61 promoted mesenchymal transformation through integrin αvβ5 in osteosarcoma

**Figure 3:**
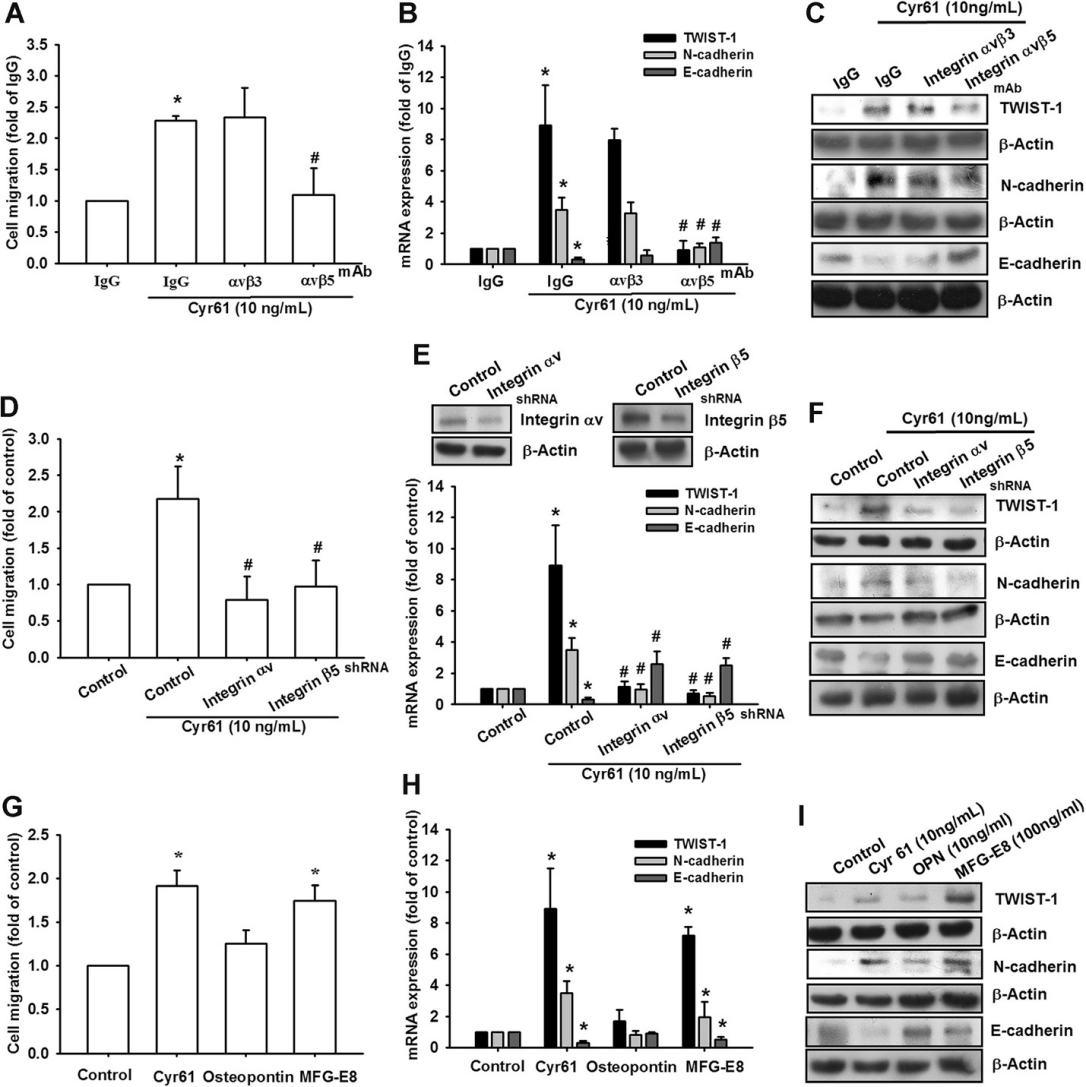
**Cyr61-promoted EMT is mediated by integrin αvβ5. (A)** MG63 cells were pretreated with anti-integrin αvβ3 or αvβ5 mAb (3 μg/mL) for 30 min, followed by stimulation with Cyr61 (10 ng/mL) for 24 h. Cell migration was measured after 24 h using the Transwell assay. The IgG served as the negative control. **(B and C)** MG63 cells were pretreated with anti-integrin αvβ3 or αvβ5 mAb (3 μg/mL) for 30 min, followed by stimulation with Cyr61 (10 ng/mL) for 24 h. Total mRNA and proteins were extracted from the MG63 cells, and the expression levels of TWIST-1, N-cadherin and E-cadherin were detected using qRT-PCR and western blotting. **(D)** MG63 cells were transfected with integrin αvβ3, αvβ5 shRNA, or control shRNA for 24 h, followed by treatment with Cyr61 (10 ng/mL) for 24 h, and cell migration was analyzed using the Transwell assay. **(E and F)** MG63 cells were transfected with integrin αvβ3, αvβ5 shRNA, or control shRNA for 24 h, followed by treatment with Cyr61 (10 ng/mL) for 24 h. Total mRNA and proteins were extracted from the MG63 cells, and expression levels of TWIST-1, N-cadherin and E-cadherin were detected using qRT-PCR and western blotting. **(G)** MG63 cells were incubated with Cyr61 (10 ng/mL), osteopontin (10 ng/mL) or MFG-E8 (100 ng/mL) for 24 h, and cell migration was measured using the Transwell assay. **(H and I)** MG63 Cells were incubated with Cyr61, osteopontin or MFG-E8 for 24 h. Total mRNA and proteins were extracted from the MG63 cells, and the expression levels of TWIST-1, N-cadherin, and E-cadherin were detected using qRT-PCR and western blotting. The results are expressed as the mean ± SEM of triplicate samples. *P <0.05 compared with the control group, and ^#^ *P* <0.05 compared with Cyr61 treatment.

Numerous reports have indicated that the CCN family interacts with integrin receptors to modulate cell biological functions [35]. Therefore, we investigated whether integrins were involved in Cyr61-promoted mesenchymal transformation in osteosarcoma. Pretreating MG63 cells with the anti-αvβ5 integrin antibody (3 μg/mL) for 30 min markedly inhibited Cyr61-induced cancer cell migration; however, the anti-αvβ3 integrin antibody did not (Figure 3A). Similarly, treating MG63 cells with the anti-αvβ5 integrin antibody inhibited the EMT markers (E-cadherin, N-cadherin, and TWIST-1) from shifting, which were regulated with Cyr61 treatment (Figure 3B-C). To confirm the role of integrin αvβ5 in Cyr61-promoted EMT in osteosarcoma, shRNAs that target integrin αv and β5 were used. The results showed that transfection with these shRNAs inhibited Cyr61-induced cell migration and the shift of EMT markers (Figure 3D-F). The authors of a previous report discussed the pivotal role of integrin αv in EMT [36]. We used MFG-E8 (a ligand of integrin αvβ5) to improve the role of integrin αvβ5 in the EMT of osteosarcoma. The result indicated that MFG-E8 induced the shift of EMT markers and cell migration ability, but osteopontin (a ligand of integrin αvβ3) did not (Figure 3G-I). In summary, these results clearly showed that Cyr61-promoted mesenchymal transformation is mediated by integrin αvβ5.

### Cyr61 promoted mesenchymal transformation via the Raf-1/MEK/ERK signaling pathway

**Figure 4:**
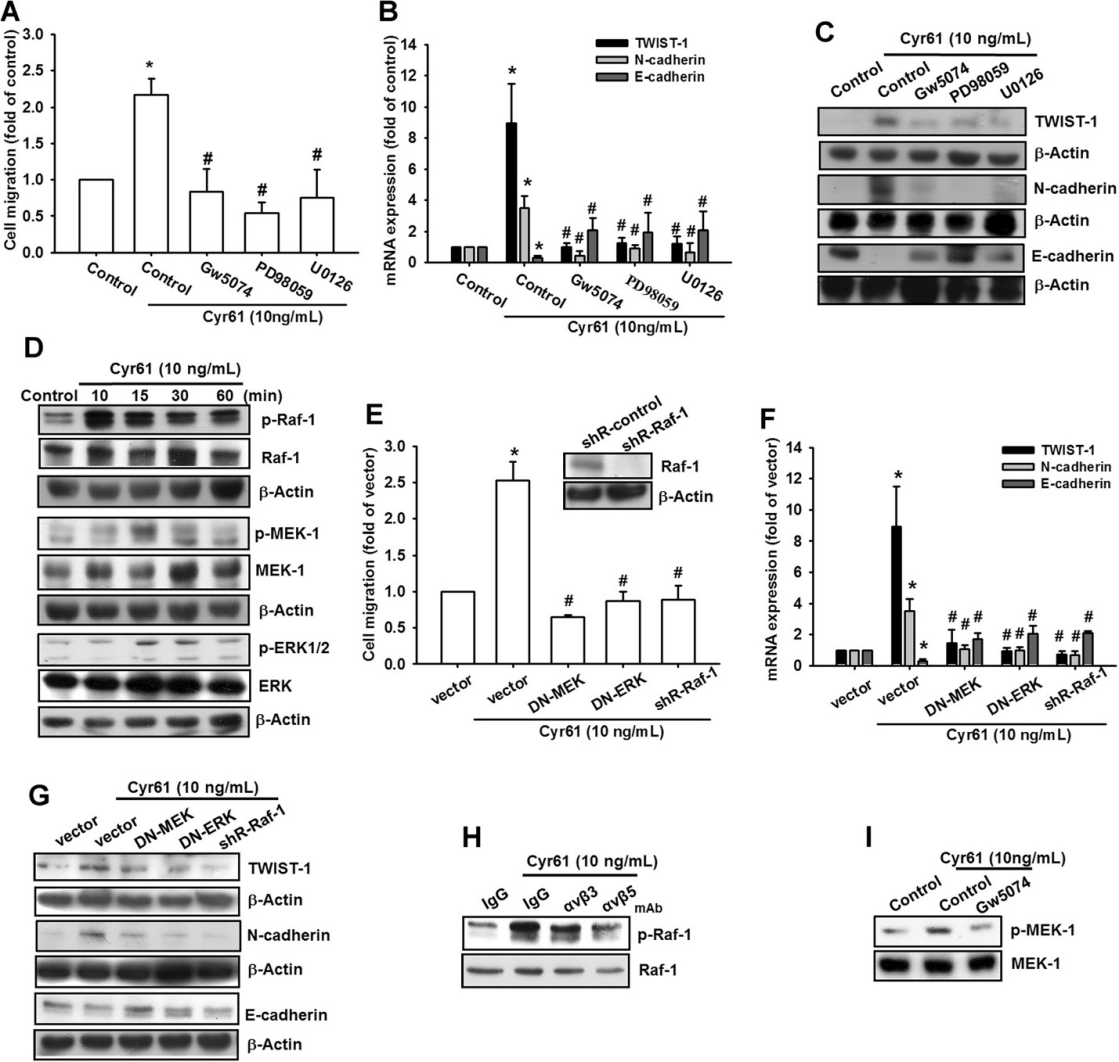
**Cyr61-promoted EMT is mediated by a Raf-1/MEK/ERK signaling cascade. (A)** MG63 cells were pretreated with Gw5074, PD98059 or U0126 for 30 min followed by stimulation with Cyr61 (10 ng/mL) for 24 h. Cell migration was measured after 24 h using the Transwell assay. **(B and C)** MG63 cells were treated as in **(A)**. Total mRNA and proteins were extracted from the MG63 cells, and the expression levels of TWIST-1, N-cadherin, and E-cadherin were detected using qRT-PCR and western blotting. **(D)** MG63 cells were incubated with Cyr61 (10 ng/mL) for the indicated times, and the phosphorylation of Raf-1, MEK, and ERK was determined through western blotting. **(E)** MG63 cells were transfected with Raf-1 shRNA, DN-MEK, DN-ERK, or a control vector for 24 h, followed by treatment with Cyr61 (10 ng/mL) for 24 h, and cell migration was analyzed using the Transwell assay. **(F and G)** MG63 cells were treated as in (E). Total mRNA and proteins were extracted from the MG63 cells, and the expression levels of TWIST-1, N-cadherin, and E-cadherin were detected using qRT-PCR and western blotting. **(H)** MG63 cells were pretreated with anti-integrin αvβ3 or αvβ5 mAb (3 μg/mL) for 30 min, followed by stimulation with Cyr61 (10 ng/mL) for 10 min, and Raf-1 phosphorylation was determined with western blotting. **(I)** MG63 cells were pretreated with Raf-1 inhibitor (Gw5074) (5 μM) for 30 min, followed by stimulation with Cyr61 (10 ng/mL) for 30 min, and MEK phosphorylation was determined with western blotting. The results are expressed as the mean ± SEM of triplicate samples. *P <0.05 compared with the control group, and ^#^ *P* <0.05 compared with Cyr61 treatment.

Previous studies have demonstrated that Ras/Raf-1/ERK signaling plays a crucial role in EMT regulation in several cancers [37–39]. Therefore, we evaluated the role of Raf-1/MEK/ERK signaling in Cyr61-promoted mesenchymal transformation. Our results indicated that pretreating MG63 cells with a Raf-1 inhibitor (Gw5074), MEK inhibitor (PD98059), and ERK inhibitor (U0126) dramatically reduced Cyr61-induced cell migration and the shift of EMT markers (Figure 4A-C). Moreover, treating MG63 cells with Cyr61 induced the phosphorylation of Raf-1, MEK, and ERK in a time-dependent manner (Figure 4D). In addition, transfecting MG63 cells with Raf-1 shRNA, dominant-mutant MEK (DN-MEK), and dominant-mutant ERK (DN-ERK) inhibited Cyr61-mediated cell migration and the shift of EMT markers (Figure 4E-G). Furthermore, pretreating of cells with the anti-αvβ5 integrin antibody reduced Cyr61-induced Raf-1 phosphorylation (Figure 4H), and pretreating cells with a Raf-1 inhibitor (Gw5074) reduced Cyr61-induced MEK phosphorylation (Figure 4I). Therefore, these results revealed that αvβ5 integrin, Raf-1, MEK, and ERK were involved in Cyr61-promoted mesenchymal transformation.

### Cyr61 promoted mesenchymal transformation via the Elk-1 signaling pathway

**Figure 5:**
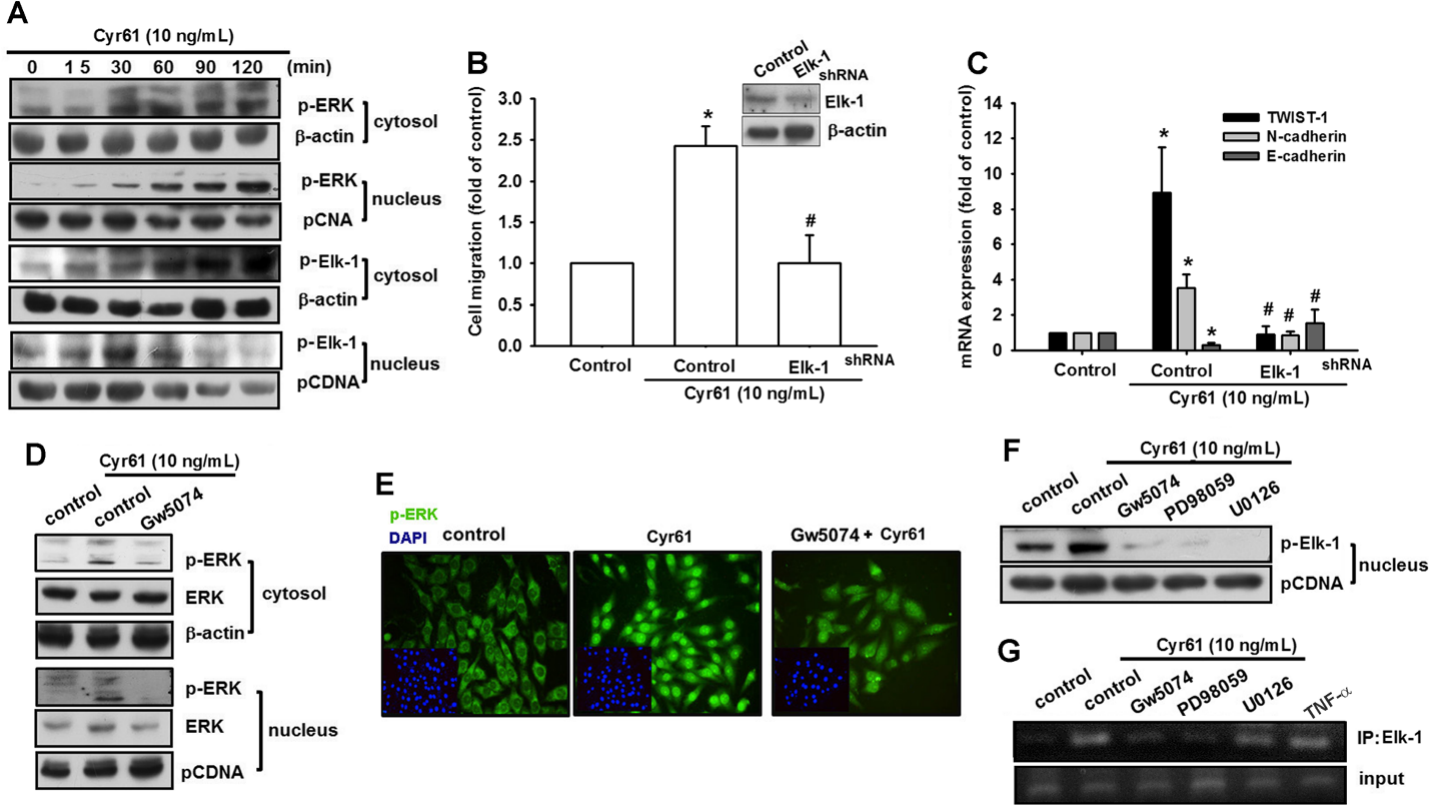
**Cyr61-promoted EMT is mediated by Elk-1 transcription factor. (A)** MG63 cells were incubated with Cyr61 (10 ng/mL) for the indicated times, the nuclear and cytosol extract were prepared, and the phosphorylation of ERK and Elk-1 was determined with western blotting. **(B)** MG63 cells were transfected with Elk-1 shRNA, or a control vector for 24 h, followed by treatment with Cyr61 for 24 h, and cell migration was analyzed using the Transwell assay. **(C)** MG63 cells were treated as in **(B)**. Total proteins were extracted from the MG63 cells, and the expression levels of TWIST-1, N-cadherin, and E-cadherin were detected with western blotting. **(D)** MG63 cells were pretreated with Gw5074 for 30 min, followed by stimulation with Cyr61 (10 ng/mL) for 60 min, and the nuclear and cytosol extract were prepared, and ERK phosphorylation was determined with western blotting. **(E)** MG63 cells were treated as in **(D)**. Cells were stained with an anti-ERK antibody and analyzed using fluorescence microscopy. Nuclei were counterstained with DAPI. Representative confocal microscopy images are shown. **(F)** MG63 cells were pretreated with Gw5074, PD98059, or U0126 for 30 min, followed by stimulation with Cyr61 (10 ng/mL) for 60 min, the nuclear extract was prepared, and Elk-1 phosphorylation was determined with western blotting. **(G)** MG63 cells were treated as in **(F)**. Chromatin immunoprecipitation was performed using anti-Elk-1. Immunoprecipitated chromatin (1%) was assayed to verify equal loading (input). The results are expressed as the mean ± SEM of triplicate samples. *P <0.05 compared with the control group and ^#^ *P* <0.05 compared with Cyr61 treatment.

The Elk-1 factor is a major downstream cytosolic transcription factor of ERK signaling, and the translocation of ERK1/2 into the nucleus is required for Elk-1 activation [40]. Our results showed that treating MG63 cells with Cyr61 increased phosphorylation and the nuclear translocation of both ERK1/2 and Elk-1 in a time-dependent manner (Figure 5A). Moreover, the transfection of MG63 cells with Elk-1 shRNA inhibited Cyr61-mediated cell migration and the shift of EMT markers (Figure 5 B&C). To confirm whether Elk-1 activation is regulated by the Raf-1/ERK signaling pathway, the Raf-1 and ERK inhibitors were used. Pretreating cells with Raf-1 inhibitors (Gw5074) reduced Cyr61-induced ERK phosphorylation and nuclear translocation (Figure 5 D&E). In addition, these inhibitors (Gw5074, PD98059 and U0126) reduced Cyr61-induced Elk-1 phosphorylation, nuclear translocation, and the binding of Elk-1 to an ETS element (Figure 5F&G). Previous studies have demonstrated that Epithelial–Mesenchymal Transition Induced by TNF-α. Therefore, we used TNF-α as a positive control for Elk ChIP assay. These results indicated that Cyr61-promoted mesenchymal transformation was mediated via the Raf-1, MEK, ERK, and Elk-1 signaling pathway.

### Knockdown of Cyr61 expression represses mesenchymal phenotype and inhibits lung metastasis in a mice model

**Figure 6:**
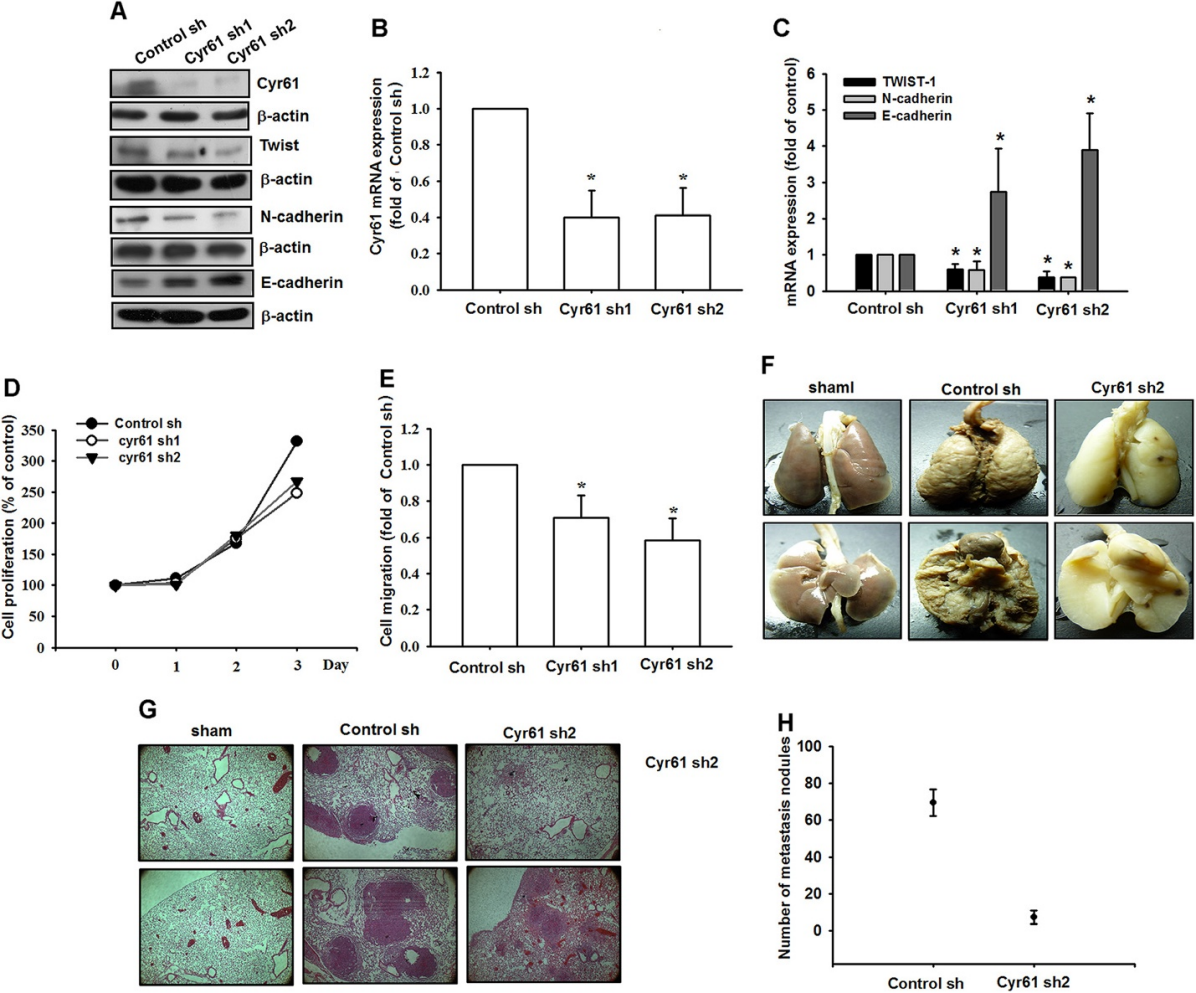
**Cyr61 knockdown reduces mesenchymal transition and lung metastasis in a mouse model. (A)** Total proteins were collected from MG63 cells stably expressing shRNAs directed against Cyr61. A vector-only control isshown (control sh). Western blot analysis was used to detect TWIST-1, N-cadherin, and E-cadherin. Actin was used as the loading control. **(B-C)** Total mRNA was collected from MG63 cells stably expressing shRNAs, and the expression levels of Cyr61, TWIST-1, N-cadherin and E-cadherin were detected using qRT-PCR. **(D)** MG63 cells stably expressing shRNA constructs were seeded as monolayers and were counted daily. Cells (1 × 10^4^) were reseeded after each count, and the cell numbers were plotted. **(E)** The *in vitro* migration of MG63 cells stably expressing shRNA constructs was measured using the Transwell assay. **(F-H)** MG63 cells stably expressing control sh and Cyr61 sh2 cells were injected into the tail vein of CB17-SCID mice. After 4 wk, the mice were sacrificed, and the lung metastatic nodules were photographed and counted. The results are expressed as the mean ± SEM of triplicate samples. *P <0.05 compared with the control group.

To confirm the role of Cyr61 in osteosarcoma lung metastasis, we exploitedMG63 and U2 OS cells that stably express Cyr61 shRNAs. After puromycin (5 μg/mL) selection, individual stable clones (Cyr61 sh1 and Cyr61 sh2) were collected for analysis. An empty vector plasmid was used as the negative control (control sh). The expression levels of Cyr61 decreased in both Cyr61 shRNA stable clones. Moreover, the expression of mesenchymal markers (TWIST-1 and N-cadherin) decreased, whereas that of the epithelial marker (E-cadherin) increased (Figure 6A-C and Additional file 1: Figure S2 A-E). The Cyr61 knockdown did not affect the cell proliferation rate (Figure 6D), but significantly reduced cell migration (Figure 6E). The lung is the most common site of osteosarcoma metastasis. Therefore, we applied axenograft mice model to determine whether Cyr61 shRNA reduced lung metastasis in MG63-injected mice *in vivo*. Obvious lung metastases were detected in the mouse lungs. By contrast, no gross metastasis were observed in mice injected with the Cyr61-knockdown MG63 (Cyr61 sh2) (Figure 6F-H). Therefore, the knockdown of Cyr61 expression repressed the mesenchymal phenotype, and inhibited lung metastasis *in vivo*.

## Discussion

Osteosarcoma is a high-grade malignant bone neoplasm that occurs mainly in children and adolescents. The chemotherapy regimens are not considerably effective, and result in the death of 20% of all patients because of lung metastasis. Therefore, developing an effective adjuvant therapy for preventing osteosarcoma metastasis is critical. EMT has received considerable attention as a conceptual paradigm for explaining invasive and metastatic behavior during cancer progression [5]. In this study, we determined that Cyr61 induced EMT and promoted lung metastasis in osteosarcoma. Moreover, Cyr61-induced EMT was mediated by an integrin αvβ5 receptor and the Raf-1, ERK, and Elk-1 signaling pathways.

In the past decade, Cyr61 has been implicated in osteo/chondrogenesis. It promotes chondrogenic differentiation through the expression of type II collagen [41]. In addition, Cyr61 expression promotes osteogenesis by increasing osteoblast differentiation and inhibiting osteoclast formation [42]. Because Cyr61 is tightly regulated in osteogenesis, aberrant levels or altered forms of CCN proteins are associated with osteosarcoma progression. The Cyr61 expression level is correlated with a poor prognosis in osteosarcoma specimens, irrespective of whether the disease is metastatic [33]. Moreover, Cyr61 expression was higher in patients with primary osteosarcoma than in those with normal bones, and was highly expressed in metastatic specimens. The knockdown of Cyr61 inhibited *in vitro* osteosarcoma cell invasion and migration as well as *in vivo* lung metastasis in mice [43]. These results showed a great opportunity for Cyr61 to be used as a novel prognosis marker and therapeutic target in osteosarcoma. Consistent with previous studies, the current study showed that Cyr61 promoted cell migration and lung metastasis through EMT in osteosarcoma (Figure 2 and 6). The Cyr61 protein promoted mesenchymal transformation by upregulating mesenchymal markers (TWIST-1 and N-cadherin) and repressing the epithelial marker (E-cadherin). This study revealed a new molecular mechanism that elucidates the role of Cyr61 in osteosarcoma progression.

Integrin is the most cruciall cell surface regulator that links the extracellular matrix to intracellular signaling molecules, and could regulate numerous cellular biological functions such as cell adhesion, signaling, motility, survival, gene expression, growth, and differentiation. Most studies have shown that Cyr61 exerts its function by directly binding to integrins [18, 44]. In this study, Cyr61-induced mesenchymal transformation was mediated by integrin αvβ5, but not by αvβ3. Moreover, treating osteosarcoma cells with the anti-αvβ5 integrin antibody inhibited Cyr61-induced mesenchymal transformation (Figure 2). The integrin αvβ3 and αvβ6 antibodies have been implicated in lung and oral cancers [45, 46]. However, this paper is the first to present a discussion on the role of integrin αvβ5 in EMT.

Several intracellular signaling proteins such as the receptor tyrosine kinase family [47], small GTPase family, and MAPK family [48] are implicated in the process of EMT. In the MAPK family, ERK, JNK, and p38 promote EMT by repressing the expression of E-cadherin through distinct mechanisms [49–51]. In this study, we determined that Cyr61 promoted mesenchymal transformation in osteosarcoma through the Raf-1/MEK/ERK signaling cascade (Figure 4). Both pathway inhibitors and dominant mutants could reduce the Cyr61-promoted mesenchymal transformation in osteosarcoma cells. Transforming growth factor-β (TGF-β), a multifunctional cytokine regulating various cellular processes, is a well-discussed EMT inducer. The TGF-β activates various signaling proteins such as Smads, phosphatidylinositol 3-kinase, and MAPK to regulate EMT [52]. Our investigation showed that the MEK/ERK signaling pathway mediated Cyr61-induced EMT, suggesting that the other MAPK members may participate in this transition. Additional efforts will be expended to determine the detailed mechanism involved in Cyr61-induced EMT in osteosarcoma.

The Elk-1 belongs to the ETS-domain transcription factor family, which regulates cell growth, differentiation, and survival. However, recent studies have revealed that Elk-1 modulates the genes involved in transcript turnover and cell migration [53]. By regulating the genes, such as plasminogen activator-1 and metalloproteinases-2 and -9, Elk-1 plays a crucial role in cancer progression. Numerous studies have indicated that Elk-1 is involved in the progression of several cancers. The Elk-1 is required for androgen-receptor-dependent growth and the survival of prostate cancer cells [54]. The Elk-1 controls cell migration by targeting various genes [55] or by regulating cell survival [56] in breast cancer. Altough the correlation between Elk-1 and cancer progression has been strengthened, we examined the role of Elk-1 in EMT for the first time in this study. The Raf-1/MEK/ERK signaling cascades activated downstream transcription factor Elk-1, and thus, participated in Cyr61-induced EMT (Figure 5). The molecular mechanism involved in Elk-1-regulated EMT will be investigated in the future.

Cancer metastasis, the major cause of mortality in cancer patients, comprises several steps through which cells detach from the primary tumor and form a secondary tumor at a distant site [57]. Abundant evidence for EMT associated with metastasis has been provided in recent studies [7]. To determine the effect of Cyr61 on osteosarcoma progression directly, we knocked down Cyr61 expression in MG63 cells by using shRNA (Figure 6). The Cyr61 knockdown significantly reduced mesenchymal markers, induced epithelial marker expression, and inhibited migration in MG63 cells. Finally, the transition of the cell phenotype inhibited lung metastasis. These data indicated that Cyr61 plays a crucial role in osteosarcoma metastasis to the lung *in vivo*.

Because lung metastasis is the major cause in mortality of patients with late-stage osteosarcoma, identifying the tumor-related factors involved in cancer metastasis is critical. In this study, we gained new insights into the Cyr61 function and its role in osteosarcoma progression. The Cyr61 expression was correlated with cell migratory potential in osteosarcoma cells, and its upregulation promotes EMT and tumor metastasis *in vivo.* Moreover, the Cyr61-promoted mesenchymal transition was mediated by the integrin αvβ5, Raf-1, MEK, ERK, and Elk-1 signaling pathways. Our observation provides a novel opportunity for treating osteosarcoma by targeting the Cyr61 gene.

## Materials and methods

### Material

Protein A/G beads, antimouse and antirabbit IgG-conjugated horseradish peroxidase, rabbit polyclonal antibodies specific for Cyr61, TWIST-1, N-cadherin, E-cadherin, p-Raf-1, Raf-1, p-ERK, ERK, p-MEK, MEK, p-Elk, Elk, PCNA and β-Actin were purchased from Santa Cruz Biotechnology (Santa Cruz, CA, USA). Rabbit polyclonal antibodies specific for αvβ3 and αvβ5 integrin were purchased from Chemicon (Temecula, CA, USA). The recombinant human Cyr61, osteopontin and MFGE-8 were purchased from PeproTech (Rocky Hill, NJ, USA). All of the shRNAs plasmids used for gene knock down were purchased from the National RNAi Core Facility Platform (Taipei, Taiwan). All of the other chemicals were obtained from Sigma-Aldrich (St Louis, MO, USA).

### Cell culture

The human osteosarcoma cell line MG63 was purchased from the American Type Cell Culture Collection (Manassas, VA, USA). The cells were maintained in Dulbecco’s Modified Eagle’s Medium, which was supplemented with 20 mM HEPES, 10% heat-inactivated fetal bovine serum, 2 mM-glutamine, penicillin (100 U/mL), and streptomycin (100 μg/mL), at 37°C with 5% CO_2_.

To establish the Cyr61 stable knockdown MG63 cell line, Cyr61 shRNA plasmids were purchased from the National RNAi Core Facility Platform (Taipei, Taiwan). The Cyr61 shRNA plasmids were transfected with Lipofectamine 2000 (Invitrogen, Carlsbad, CA, USA) and Cyr61 shRNA-expressing cells were puromycin selected (10 μg/mL). The surviving cells were selected and expanded to produce clonal cell populations. For monolayer growth curves, 10^4^ cells were plated in 6-well plates and grown for 1–3 days. The cells were trypsinized, and the cell numbers were counted daily.

### Establishment of migration-prone sublines

The subpopulations from MG63 cells were selected according to their differential migration ability [58]; the cell culture insert system was used as described. After 24 h of migration, cells that penetrated pores and migrated to the underside of the filters were trypsinized and harvested for a second round of selection. The original cells that did not penetrate membrane pores were designated as M0. After 10 rounds of selection, the migration-prone subline was designated as M10.

### Migration assay

The migration assay was performed using the Transwell assay (Costar, NY, USA; pore size: 8 μm) in 24-well dishes. Before the migration assay, the cells were pretreated with different concentrations of inhibitors, including Gw5047, PD98059, U0126, and the vehicle control (0.1% DMSO), or neutralized antibodies such as Cyr61, αvβ3, and αvβ5 integrin, and the vehicle control (IgG) for 30 min, or they were transfected with the indicated shRNA plasmids, including the TWIST-1, integrin αv, integrin β5, Raf-1, and Elk-1 for 24 h. After pretreatment, approximately 1 × 10^4^ cells in 200 μL of a serum-free medium were placed in the upper chamber, and 300 μL of the same medium containing Cyr61, osteopontin or MFGE-8 was placed in the lower chamber. The plates were incubated for 24 h at 37°C in 5% CO_2_, and then the cells were fixed in methanol for 15 min and stained with 0.05% crystal violet in PBS for 15 min. The cells on the upper side of the filters were removed with cotton-tipped swabs, and the filters were washed with PBS. The cells on the underside of the filters were examined and counted using a microscope. Each experiment was repeated at least 3 times. The number of invading cells in each experiment was adjusted using the cell viability assay to correct for the proliferation effects of Cyr61 treatment (corrected invading cell number = counted invading cell number/percentage of viable cells).

### Wound-healing assay

For wound-healing migration assays, the cells were seeded on 12-well plates at a density of 2 × 10^5^ cells/ per well in a culture medium. At 24 h after seeding, the cells were treated with the indicated inhibitors or a neutralized antibodies for 30 min or transfected with shRNA plasmids for 24 h. After pretreatment, the confluent monolayer of the culture was scratched using a fine pipette tip, and incubated with recombinant Cyr61 for 24 h and migration was observed using microscopy. The rate of wound closure was observed at the indicated times.

### Quantitative real-time polymerase chain reaction

Quantitative real-time polymerase chain reaction (qRT-PCR) analysis was performed using Taqman® one-step PCR Master Mix (Applied Biosystems, Foster City CA, USA). Furthermore, 100 ng of total cDNA was added to every 25-μL reaction with sequence-specific primers and Taqman® probes. The sequences for all target gene primers and probes were purchased commercially (β-actin was used as the internal control) (Applied Biosystems, Foster City CA, USA). The qRT-PCR assays were conducted in triplicate on a StepOnePlus sequence detection system. The cycling conditions were 10-min of polymerase activation at 95°C, followed by 40 cycles at 95°C for 15 s and 60°C for 60 s. The threshold was set higher than the non-template control background and within the linear phase of target gene amplification for calculating the cycle number at which the transcript was detected (denoted as C_T_).

### Western blot analysis

The celllysates were prepared, and proteins were then resolved on sodium dodecyl sulfate-polyacrylamide gel electrophoresis, and transferred to Immobilon polyvinyldifluoride membranes. The blots were blocked with 4% BSA for 1 h at room temperature and then probed with rabbit antihuman antibodies against Cyr61, TWIST-1, N-cadherin, E-cadherin, p-Raf-1, Raf-1, p-ERK, ERK, p-MEK, MEK, p-Elk, and β-Actin (1:1000) for 1 h at room temperature. After 3 washes, the blots were subsequently incubated with a donkey antirabbit peroxidase-conjugated secondary antibody (1:1000) for 1 h at room temperature. The blots were observed using enhanced chemiluminescence by employing Kodak X-OMAT LS film (Eastman Kodak, Rochester, NY, USA). Quantitative data were obtained using a computing densitometer and ImageQuant software (Molecular Dynamics, Sunnyvale, CA, USA).

Nuclear extracts were prepared as described previously [58]. The cells were suspended in buffer A for 10 min on ice. The lysates were separated into cytosolic and nuclear fractions through centrifugation at 12000 g for 10 min. The supernatants containing cytosolic proteins were collected. A pellet containing nuclear fractions was resuspended in buffer C for 30 min on ice. The supernatants containing nuclear proteins were collected through centrifugation at 13 000 g for 20 min, and stored at -80°C.

### Chromatin immunoprecipitation assay

Chromatin immunoprecipitation analysis was performed as described previously [59]. DNA immunoprecipitated using the anti-Elk antibody was purified. The DNA was then extracted using phenol–chloroform. The purified DNA pellet was subjected to PCR, and the PCR products were then resolved by conducting 1.5% agarose gel electrophoresis, and they were observed using ultraviolet. The primers 5′-AGCCCCAGCAATCCAAATC-3′ and 5′-TCGGAGGAGACTGTCCTGG-3′ were used for amplification across the human TWIST-1 promoter region (1466 to 1613).

### Immunofluorescence microscopy

The MG63 cells grown on glass coverslips were rinsed once with PBS, and fixed in 3.7% paraformaldehyde for 10 min in RT. The cells were then washed 3 times with PBS, and blocked with 4% BSA for 15 min. The cells were then incubated with rabbit antimouse Elk (1:100) for 1 h in RT, rewashed, and incubated with FITC-conjugated goat anti-rabbit IgG for 1 h. Finally, the cells were washed, mounted, and examined using a Leica TCS SP2 Spectral Confocal System.

### In vivo tumor xenograft study

Four-week-old male SCID mice were purchased from Lasco (Taipei, Taiwan) and maintained under pathogen-free conditions. All of the animal experiments were performed according to a protocol approved by the Shin Kong Wu Ho-Su Memorial Hospital (Taipei, Taiwan) Institutional Animal Care and Use Committee. Male CB17/SCID mice (4 wk old) were used. Seven animals per group, were used, and the experiment was repeated twice. For experimental metastasis assays, 1 × 10^6^ cells were resuspended in 0.1 mL of PBS, and injected into the lateral tail vein. After 4 wk, the mice were euthanized using an overdose of the anesthetic agent. The lungs were removed and fixed in 10% formalin. The number of lung tumor metastases was counted using a dissecting microscope.

### Statistical analysis

Data are presented as the mean ± standard error of the mean (SEM). Statistical comparisons between two samples were performed using Student’s *t* test. Statistical comparisons of more than 2 groups were performed using one-way analysis of variance with Bonferroni’s post hoc test. A *P*-value less than 0.05 was considered statistically significant.

## Electronic supplementary material

Additional file 1: **Cyr61 expression level in human fetal osteoblastic cell line compared with osteosarcoma cell lines.** **Figure S1.** The expression of Cyr61 in human osteosarcoma cells. (A-B) Total protein and mRNA were extracted form hFOB 1.19, U2 OS, and U2OS cells, the Cyr61 expression was examined by western blot analysis and qPCR. Results are expressed as the mean ± SEM. *p < 0.05 compared with hFOB 1.19. Knockdown of Cyr61 expression represses mesenchymal phenotype and inhibits cell migration in U-2 OS cell line. **Figure S2.** Cyr61 knockdown reduces mesenchymal transition in U2 OS cell lins. (A) Total proteins were collected from U2 OS cells stably expressing shRNAs directed against Cyr61. A vector-only control is shown (control sh). Western blot analysis was used to detect Cyr61, TWIST-1, N-cadherin, and E-cadherin. Actin was used as the loading control. (B-C) Total mRNA was collected from U2 OS cells stably expressing shRNAs, and the expression levels of Cyr61, TWIST-1, N-cadherin and E-cadherin were detected using qRT-PCR. (D) U2 OS cells stably expressing shRNA constructs were seeded as monolayers and were counted daily. Cells (1 × 10^4^) were reseeded after each count, and the cell numbers were plotted. (E) The *in vitro* migration of U2 OS cells stably expressing shRNA constructs was measured using the Transwell assay. Results are expressed as the mean ± SEM. *p < 0.05 compared with control sh. (DOCX 189 KB)

## Abbreviations

(EMT): Epithelial to mesenchymal transition
(Cyr61): cysteine-rich angiogenic inducer 61
(CTGF/CCN2): connective tissue growth factor
(Nov/CCN3): nephroblastoma overexpressed
(CCN4): Wisp-1/elm1
(CCN5): Wisp-2/rCop1
(CCN6): Wisp-3
(MEK): Mitogen activated protein kinase kinase
(ERK): Extracellular signal-regulated kinase
(DN): Dominant-mutant
(RTK): Receptor tyrosine kinase
(TGF-β): Transforming growth factor-β
(PI3K): Phosphatidylinositol 3-kinase
(PAI-1): Plasminogen activator-1
(MMPs): Metalloproteinases
(shRNAs): Short hairpin RNAs
(qRT-PCR): Quantitative real-time polymerase chain reaction
(PVDF): Polyvinyldifluoride
(ChIP): Chromatin immunoprecipitation analysis.
