# Supplementary material for: Cyr61 promotes epithelial-mesenchymal transition and tumor metastasis of osteosarcoma by Raf-1/MEK/ERK/Elk-1/TWIST-1 signaling pathway
Source: Mol Cancer. 2014 Oct 19;13:236. doi: 10.1186/1476-4598-13-236 (PMC4210521; doi:10.1186/1476-4598-13-236)
Supplement: Supplementary file 1 — Additional file 1: Cyr61 expression level in human fetal osteoblastic cell line compared with osteosarcoma cell lines. Figure S1. The expression of Cyr61 in human osteosarcoma cells. (A-B) Total protein and mRNA were extracted form hFOB 1.19, U2 OS, and U2OS cells, the Cyr61 expression was examined by western blot analysis and qPCR. Results are expressed as the mean ± SEM. *p < 0.05 compared with hFOB 1.19. Knockdown of Cyr61 expression represses mesenchymal phenotype and inhibits cell migration in U-2 OS cell line. Figure S2. Cyr61 knockdown reduces mesenchymal transition in U2 OS cell lins. (A) Total proteins were collected from U2 OS cells stably expressing shRNAs directed against Cyr61. A vector-only control is shown (control sh). Western blot analysis was used to detect Cyr61, TWIST-1, N-cadherin, and E-cadherin. Actin was used as the loading control. (B-C) Total mRNA was collected from U2 OS cells stably expressing shRNAs, and the expression levels of Cyr61, TWIST-1, N-cadherin and E-cadherin were detected using qRT-PCR. (D) U2 OS cells stably expressing shRNA constructs were seeded as monolayers and were counted daily. Cells (1 × 10^4^) were reseeded after each count, and the cell numbers were plotted. (E) The in vitro migration of U2 OS cells stably expressing shRNA constructs was measured using the Transwell assay. Results are expressed as the mean ± SEM. *p < 0.05 compared with control sh. (DOCX 189 KB) [file 12943_2014_1436_MOESM1_ESM.docx]

**Supplementary data**

**

**

**Fig. S1: The expression of Cyr61 in human osteosarcoma cells.** (A-B) Total protein and mRNA were extracted form hFOB 1.19, U2 OS, and U2OS cells, the Cyr61 expression was examined by western blot analysis and qPCR. Results are expressed as the mean ± SEM. *, p < 0.05 compared with hFOB 1.19.

;



**Fig. S2: Cyr61 knockdown reduces mesenchymal transition in U2 OS cell lins.** (A) Total proteins were collected from U2 OS cells stably expressing shRNAs directed against Cyr61. A vector-only control is shown (control sh). Western blot analysis was used to detect Cyr61, TWIST-1, N-cadherin, and E-cadherin. Actin was used as the loading control. (B-C) Total mRNA was collected from U2 OS cells stably expressing shRNAs, and the expression levels of Cyr61, TWIST-1, N-cadherin and E-cadherin were detected using qRT-PCR. (D) U2 OS cells stably expressing shRNA constructs were seeded as monolayers and were counted daily. Cells (1 x 10^4^) were reseeded after each count, and the cell numbers were plotted. (E) The *in vitro* migration of U2 OS cells stably expressing shRNA constructs was measured using the Transwell assay. Results are expressed as the mean ± SEM. *, p < 0.05 compared with control sh.
